# Nickel isotopes link Siberian Traps aerosol particles to the end-Permian mass extinction

**DOI:** 10.1038/s41467-021-22066-7

**Published:** 2021-04-01

**Authors:** Menghan Li, Stephen E. Grasby, Shui-Jiong Wang, Xiaolin Zhang, Laura E. Wasylenki, Yilun Xu, Mingzhao Sun, Benoit Beauchamp, Dongping Hu, Yanan Shen

**Affiliations:** 1 School of Earth and Space Sciences, University of Science and Technology of China, Hefei, China; 2 Geological Survey of Canada, Natural Resources Canada, Calgary, Alberta Canada; 3 Department of Geoscience, University of Calgary, Calgary, Alberta Canada; 4 State Key Laboratory of Geological Processes and Mineral Resources, China University of Geosciences, Beijing, China; 5 School of Earth & Sustainability, Northern Arizona University, Flagstaff, AZ USA; 6 Department of Earth Sciences, ETH Zürich, Zürich, Switzerland

**Keywords:** Geochemistry, Volcanology

## Abstract

The end-Permian mass extinction (EPME) was the most severe extinction event in the past 540 million years, and the Siberian Traps large igneous province (STLIP) is widely hypothesized to have been the primary trigger for the environmental catastrophe. The killing mechanisms depend critically on the nature of volatiles ejected during STLIP eruptions, initiating about 300 kyr before the extinction event, because the atmosphere is the primary interface between magmatism and extinction. Here we report Ni isotopes for Permian-Triassic sedimentary rocks from Arctic Canada. The δ^60^Ni data range from −1.09‰ to 0.35‰, and exhibit the lightest δ^60^Ni compositions ever reported for sedimentary rocks. Our results provide strong evidence for global dispersion and loading of Ni-rich aerosol particles into the Panthalassic Ocean. Our data demonstrate that environmental degradation had begun well before the extinction event and provide a link between global dispersion of Ni-rich aerosols, ocean chemistry changes, and the EPME.

## Abstract (web summary)

The end-Permian mass extinction was the most severe extinction event in the past 540 million years, and the Siberian Traps large igneous province is widely hypothesized to have been the primary trigger for the environmental catastrophe. In this study, Ni isotopes provide the link between Siberian Traps magmatism and early environmental degradation, ultimately leading to the end-Permian extinction.

## Introduction

The end-Permian mass extinction (EPME) about 252 million years ago (Ma) was the most severe biotic crisis in the Phanerozoic, eliminating more than 90% of marine and 75% of terrestrial species^1^. The Siberian Traps large igneous province (STLIP), the largest known continental flood basalt province, is widely hypothesised to have been the primary trigger for the catastrophic environmental deterioration driving the EPME^2–6^. Potential kill mechanisms triggered by emplacement of the Siberian Traps magmas include global warming, ultraviolet radiation exposure, hypercapnia, ocean acidification and anoxia, and toxic metal release^4–14^. All of these potential mechanisms are critically dependent on the exact nature and timing of volatiles ejected during eruption because the atmosphere is the primary interface between STLIP magmatism and extinction. The initial eruption of the STLIP was about 300 ± 126 kyr prior to the onset of the EPME in marine realms^3^, during which the emplacement of the enormous Noril’sk nickel sulphide ore deposits in the Tunguska Basin may have released voluminous nickel-rich volcanic gas and aerosols into the atmosphere^15^. A spike in Ni abundance at the extinction level in the Meishan section, South China, is hypothesised to have triggered an explosive expansion of methanogenic Archaea and exponential increase of the marine inorganic carbon reservoir, leading to catastrophic mass extinction^16^. However, the Ni enrichment at Meishan could have also resulted from diagenesis^17^, and therefore the origin of that Ni spike needs to be further evaluated^16^. In addition, little is known about the potential timing of Ni loading to the oceans and how voluminous release and dispersal of Ni-rich aerosols may have impacted ocean chemistry, climate change, and ultimately the EPME. Here, we report Ni isotopes (δ^60^Ni) and Ni abundances for the Permian-Triassic sedimentary rocks from the Buchanan Lake section in the Sverdrup Basin, Canadian High Arctic. We use Ni isotopic and elemental data to study the climate and environmental consequences of emissions of Ni-rich aerosols and present a new scenario that links atmospheric and ocean chemistry changes triggered by the STLIP to the EPME.

## Results and discussion

### Geological setting and sampling

**Fig. 1:**
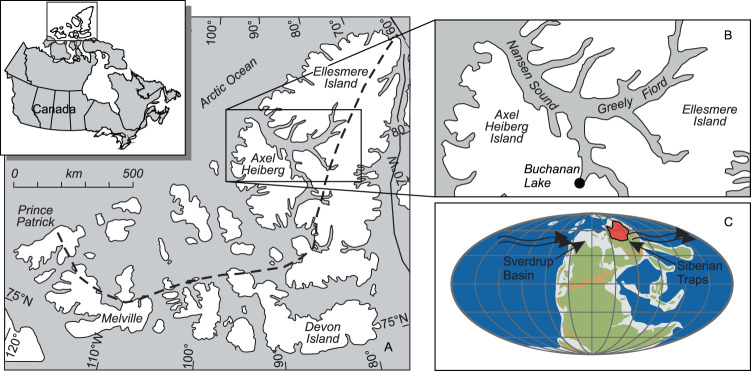
Regional map showing the location of the study area in Arctic Canada (after ref. ^22^). **A** Regional map. **B** Detailed map showing the location of the Buchanan Lake section. **C** Permian palaeogeographic map showing the location of the Sverdrup Basin relative to the Siberian Traps volcanic rocks (base map after C.R. Scotese [http://www.scotese.com/]). Wavy arrows indicate predominant westerly wind patterns.

**Fig. 2:**
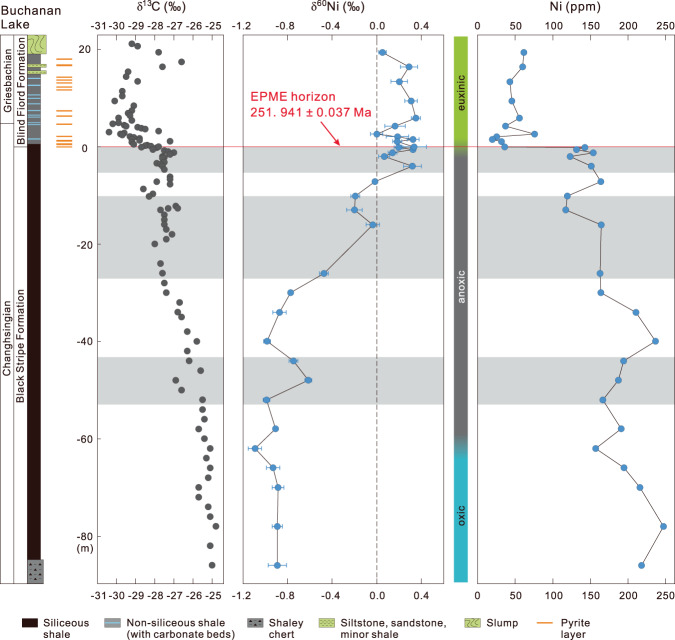
Nickel isotopic compositions and Ni contents of black shales in the Buchanan Lake section. Error bars represent 2 s.d. The C-isotope chemostratigraphy is from ref. ^5^ and shaded areas refer to fly coal ash loading events reported in ref. ^5^. Palaeo-redox conditions are from refs. ^21,22^ and U-Pb age for the EPME horizon is from ref. ^27^.

The Sverdrup Basin was a Carboniferous to Paleogene depocenter that accumulated over 12 km of sediment from Carboniferous to Paleogene time^18^ (Fig. 1). From Late Carboniferous to Early Triassic time, the Sverdrup Basin was along the NW margin of Pangea at palaeolatitudes of 35–40°N (ref. ^19^) (Fig. 1). Until the EPME, the basin was characterised by a central deep basinal area of fine-grained clastic deposition surrounded by a shallow shelf dominated by biogenic carbonate that transitioned in the late Permian to chert formed by shallow water siliceous sponges^19^. After the EPME, the Sverdrup basin was dominated by clastic-dominated sedimentation^18^. In this study, we examined the distal deep-water Buchanan Lake section which preserves outstanding Boreal records of the EPME, followed by the biotic recovery in the Early Triassic^5^. The Buchanan Lake section consists mostly of black shale of the Late Permian Black Stripe Formation and overlying Early Triassic Blind Fiord Formation that preserves characteristic post-extinction fauna^20^ (Fig. 2).

During the last decade, the Buchanan Lake section has been extensively examined, and the carbon isotope chemostratigraphy, elemental compositions of the shale, and oceanic palaeo-redox changes have been well constrained^5,11,19–26^ (Fig. 2). The EPME in the Sverdrup Basin is marked by eradication of silica and carbonate producers along with the onset of a significant negative δ^13^C_org_ shift that has been correlated globally with the dated Global Stratotype Section and Point (GSSP) for the Permian-Triassic boundary at Meishan, China, at ~251.9 Ma (refs. ^3,4,20,27,28^) (Fig. 2). The palaeo-redox conditions during the deposition of the Late Permian Black Stripe Formation and Early Triassic Blind Fiord Formation evolved from an oxic water column with a strong redoxcline in the sediments to anoxic and then to sulphidic bottom water conditions (Fig. 2). The average total sulphur (TS) content for the samples from the oxic interval of the Black Stripe Formation (−86 m to −62 m) is 0.50 wt%, and the samples from the anoxic interval of the Black Stripe Formation (−62 to −2 m) have an average TS content of 0.89 wt%, except for one sample (C-445100) having TS content of 12.27 wt% (ref. ^21^). The sulphidic shales of the uppermost Black Stripe Formation are characterised by high TS contents with an average TS content of 2.09 wt% and small grains of framboidal pyrite^5,21^. The sulphidic shales of the lower Blind Fiord Formation have an average TS content of 1.15 wt% and contain pyrite layers of ~1 cm in thickness^5,21^ (Fig. 2).

### Nickel isotopic and content data from the Buchanan Lake section

Nickel isotopic compositions (δ^60^Ni) are reported in delta notation relative to the Ni isotopic standard NIST SRM 986, in units of per mil (‰): [δ^60/58^Ni = (^60^Ni/^58^Ni_sample_/^60^Ni/^58^Ni_SRM986_ − 1) × 1000]. The δ^60^Ni compositions and Ni contents of the Buchanan Lake samples are presented in Fig. 2 (full analytical data are available as Supplementary Table 1). The δ^60^Ni and Ni contents show distinctive variations from pre-extinction to syn-extinction to post-extinction (Fig. 2).

Prior to the EPME, the δ^60^Ni values for the black shale (−86 to −62 m) deposited predominantly under oxic bottom water conditions show significantly light δ^60^Ni values ranging from −0.89‰ to −1.09‰, with Ni contents from 157.1 to 247.1 ppm (Fig. 2). The δ^60^Ni values for the black shale (−62 to −2 m) of the Black Stripe Formation deposited under anoxic bottom water conditions display a general increase from −0.99 to 0.32‰, although a minor variation up to 0.37‰ from −50 to −40 m is observed (Fig. 2). During the same interval, Ni contents vary between 117.5 and 247.1 ppm, although only a weak stratigraphic trend is exhibited, with decreasing Ni over time (Fig. 2).

The δ^60^Ni values for the black shale (−2 to 0 m) deposited under anoxic and sulphidic bottom water conditions of the Black Stripe Formation exhibit positive δ^60^Ni values from 0.07‰ to 0.34‰, and δ^60^Ni does not change significantly at the extinction horizon (Fig. 2). However, the Ni content drops sharply from 142.8 to 36.4 ppm at this level (Fig. 2). After the extinction, the δ^60^Ni values for the sulphidic black shale of the lower Blind Fiord Formation (0–19.4 m) vary between 0.00 and 0.34‰, and the Ni contents vary between 25.5 and 61.7 ppm (Fig. 2).

### Nickel cycling and isotopic record in modern depositional systems

**Fig. 3:**
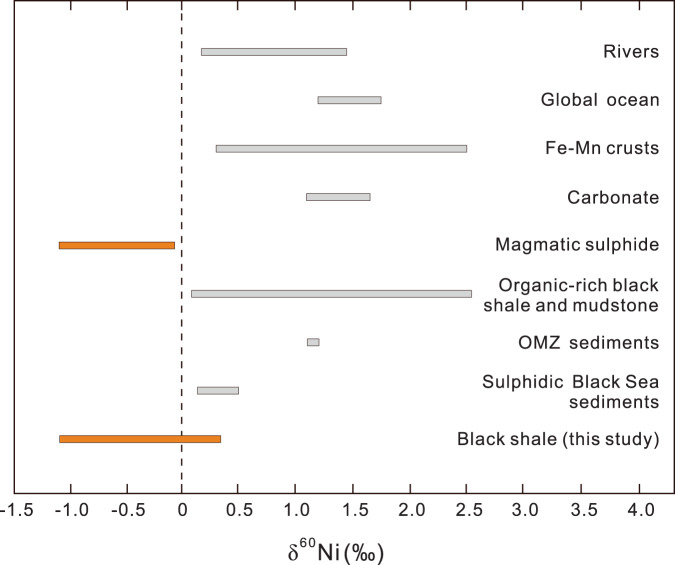
Summary of the Ni isotopic compositions reported in this study and previously published data. Rivers and the global ocean are from ref. ^36^, and Fe–Mn crusts are from refs. ^35,45^. Carbonates and OMZ sediments are from ref. ^43^. Magmatic sulphides are from refs. ^38,48^. Organic-rich black shale and mudstone are from ref. ^47^ and sulphidic Black Sea sediments are from ref. ^33^. The comparable light Ni isotopic compositions between the magmatic sulphides and our data are highlighted in orange.

To evaluate possible interpretations of the Ni concentration and isotope profile in the Buchanan Lake section, we present a brief review of the Ni cycle and summarise the δ^60^Ni data that are closely relevant to our study (Fig. 3). Nickel is a bioessential trace metal, and, like phosphate and silica in the modern ocean, Ni exhibits a nutrient-type profile, with low concentrations in the photic zone due to biological uptake, and high concentrations at greater water depths due to the recycling of organic matter in the water column^29^. Nickel is also an essential component of important enzymes for the metabolism of methanogens and has been shown to have played a critical role in methane production and atmospheric oxygenation in the Archaean^30–32^. Like other stable isotope systems, the application of δ^60^Ni compositions to reconstruct Earth history depends on our understanding of the Ni cycling and δ^60^Ni variations in modern rivers, oceans, and sediments^32–34^. Dissolved Ni in modern oceans is mainly from: (1) weathering products of the continental crust transported by rivers to the oceans, (2) mineral dust and volcanogenic aerosols settling to the oceans from the atmosphere, and (3) hydrothermal vent fluids as a minor source^34–37^. The δ^60^Ni of dissolved Ni from riverine influx to the oceans has a heavy isotopic composition with an average δ^60^Ni value of +0.84‰ (ref. ^36^) (Fig. 3). The enrichment in heavy isotopes of riverine Ni relative to continental source rocks (δ^60^Ni mostly between −0.1‰ and +0.2‰)^38^ is likely caused by sorption of light Ni onto Fe oxides and authigenic clays during weathering^37,39–42^. Modern seawater has an average δ^60^Ni value of 1.44 ± 0.015‰, which is considerably heavier than the riverine inputs to the oceans^36,43,44^ (Fig. 3).

The modern outputs of Ni from the ocean reservoir include sediments deposited under various redox conditions, as well as Fe–Mn crusts. Hydrogenic Fe–Mn crusts that potentially record the Ni isotopic composition of deep ocean water have a wide range of heavy δ^60^Ni values, from 0.25 to 2.5‰ with an average δ^60^Ni = 1.74 ± 0.59‰ (*n* = 126)^35,45^ (Fig. 3). The δ^60^Ni values for most of the sediments deposited either under oxic, suboxic, or anoxic conditions are similar to, or heavier than, seawater^43^. Therefore, at present, the budget of Ni isotopes at steady-state between inputs and outputs has an apparent imbalance^35–37,43,45^. However, δ^60^Ni values ranging from −0.2‰ to −0.8‰ were reported from the sediments that were deposited under oxygenated bottom water conditions at water depths of >3 km in the eastern Pacific^46^. These light δ^60^Ni values are interpreted to have resulted from diagenetic remobilisation, or mineralogical transformation of birnessite to todorokite, or possible scavenging of light Ni by Fe oxides or Fe-rich authigenic clays^46^.

We note that δ^60^Ni data from the oxygen minimum zone (OMZ) and the Black Sea sediments may be important in the interpretation of δ^60^Ni values in the rock record, especially in relation to ocean anoxic events that have occurred throughout Earth’s history. The δ^60^Ni values from Peru Margin OMZ sediments are between 1.11 and 1.21‰, with an average value of 1.16‰ (ref. ^43^) (Fig. 3). The Black Sea sulphidic sediments are characterised by lighter δ^60^Ni compositions ranging from 0.14 to 0.51‰ (Fig. 3), compared to the δ^60^Ni of 1.26‰ for dissolved Ni of the Danube River that enters the Black Sea^33^.

Regarding the geologic rock record, the only detailed δ^60^Ni studies so far are those of the organic-rich black shale sequence from the Jurassic Sinemurian-Pliensbachian GSSP at Robin Hood’s Bay, UK, and the Devonian–Mississippian Exshaw Formation in the West Canada Sedimentary Basin^47^ (Fig. 3). These black shales show δ^60^Ni values varying widely from 0.2 to 2.5‰, with some significantly lighter and some heavier than modern seawater’s homogeneous δ^60^Ni value^47^. These large variations in δ^60^Ni values may record variability in Ni sources to the oceans^47^. It is worth noting that the lightest δ^60^Ni values reported so far are from the Ni–sulphide ores hosted by Archaean komatiites which have δ^60^Ni compositions from −0.10 to −1.03‰ (mostly from −0.62 to −1.03‰)^38^ (Fig. 3). Similar light δ^60^Ni compositions of −0.82 ± 0.02‰ were reported from the Archaean Ni-rich magmatic sulphides^48^ (Fig. 3). These exceptionally light δ^60^Ni values likely reflect Ni isotopic fractionation during magmatic Ni sulphide formation or melting at high temperature^38,48^.

### Environmental significance of the Buchanan Lake data

The δ^60^Ni values of black shales from the Buchanan Lake section span a very wide range, from −1.09 to 0.35‰ (Fig. 2). The lightest δ^60^Ni values occur in the oxic interval of the Black Stripe Formation, heavier values toward the anoxic interval of the upper Black Stripe Formation, and slightly heavier in the euxinic interval, suggesting a strong contrast with the isotopic homogeneity of the present-day seawater (Figs. 2 and 3). The δ^60^Ni values in the lower Black Stripe Formation are among the lightest ever reported for sedimentary rocks; the only reported lighter values are those from magmatic Ni sulphide deposits^38,48^ (Fig. 3). Of all the known mechanisms potentially responsible for the overall light δ^60^Ni values from the Buchanan Lake section, the most convincing is pre-EPME input to Sverdrup Basin of Ni sourced from voluminous Ni-rich aerosols released during the STLIP emplacement of nickel sulphide ore deposits. Other explanations for the light δ^60^Ni values are difficult to support. For example, fractionation during sorption to Mn oxyhydroxide particle surfaces might result in the deposition of isotopically light Ni from seawater. Large Ni isotopic fractionations of up to −3.35‰ caused by surface complexation reactions between the mineral and aqueous phases were reported in laboratory sorption experiments with hexagonal birnessite^49^. However, the difference between the Late Permian pyritic black shales and Mn oxides indicate that Ni sorption onto MnO_2_ cannot explain the light δ^60^Ni values in the black shales. Alternatively, continental weathering of magmatic Ni sulphides could potentially transport relatively light δ^60^Ni to the oceans compared to the average continental crust. However, there is no occurrence of magmatic Ni–sulphide ores in the Sverdrup Basin and surrounding margins that could be a primary contributor^50^. Although no Ni isotopic data are available for Late Permian marine hydrothermal fluids, it is unlikely that such fluids would have differed much in δ^60^Ni throughout the Phanerozoic Eon; there is no reason to expect short-lived changes that would have caused the exceptionally light δ^60^Ni values in the Buchanan Lake shales. Regardless, there is no evidence of hydrothermal activities in the Sverdrup Basin that could be a source of Ni^50^.

Several lines of evidence support our interpretation that isotopically light, Ni-rich aerosols came to be a dominant source of Ni input to the Late Permian Panthalassic Ocean. Nickel concentrations in the Black Stripe Formation samples (117.5–247.1 ppm) are well above the 68 ppm Ni for average shale^51^ (Fig. 2). This Ni enrichment is contemporaneous with episodic coal ash fallout and Hg deposition into the basin, as well as the initial onset of decline in δ^13^C values (Fig. 2), previously tied to early eruption phases of the Siberian Traps^5,11^. The age model, based on sedimentation rate, suggests that early onset eruption recorded at Buchanan Lake started ~500 ky prior to the EPME^5,11^. This timing is remarkably consistent with the initial emplacement of the vertical dike-sill system of the STLIP which is preceded the onset of EPME by at least 300 ± 126 ky (ref. ^4^). Additional support for a volcanic source comes from the Ni concentration profile recorded in the Noril’sk lava stratigraphy, which shows high Ni concentrations prior to the EPME and a significant drop when the EPME occurred^3^, as observed in the Buchanan Lake section.

We suggest that the melting and degassing of Ni sulphide during emplacement processes^15^ may have produced light δ^60^Ni compositions as a signature of the Siberian Traps, similar to the light δ^60^Ni compositions measured from magmatic Ni–sulphide (Fig. 3). The Noril’sk ore deposits may be the only known occurrence of a flood basalt-associated magmatic sulphide system that was shallow enough to degas^15^. Nickel is nonvolatile and normally locked up at depth in magmatic minerals. However, Ni scavenged by magmatic sulphides could have been transferred to magmatic gases, and flotation of Ni-rich sulphide droplets to the surface by gas bubbles may have produced voluminous Ni-rich aerosols^15^. The global dispersion of Ni-rich aerosols and loading into the Sverdrup Basin, ~20,000 km downwind of the Siberian Traps^5^, would have been rapid (about 4–8 days) during stratospheric eruptions. The fallout of volcanic Ni emissions would have significantly changed the Ni-isotopic compositions of seawater, resulting in the observed light δ^60^Ni values in the Black Stripe Formation (Fig. 2).

The δ^60^Ni increase of 1.23‰ from −0.91 to 0.32‰ during the anoxic interval of the Black Stripe Formation may reflect gradually decreased input of Ni-rich aerosols (Fig. 2). The sharp drop in Ni abundance near the extinction level suggests a greatly reduced loading of Ni-rich aerosols to the Sverdrup Basin during the EPME. It may also reflect the rapid removal of Ni by the expansion of methanogens which resulted in a large pool of methane to the ocean, contributing to the global negative C-isotopic excursions^16^ (Fig. 2). The sulphidic black shales of the uppermost Black Stripe Formation (−2 to 0 m) have δ^60^Ni values from 0.07 to 0.34‰, similar to the δ^60^Ni values of 0.14–0.51‰ for the sulphidic sediments in the modern Black Sea. After the extinction, comparable δ^60^Ni values of 0–0.34‰ are observed for the sulphidic black shale of the lower Blind Fiord Formation (Fig. 2). The δ^60^Ni values from the uppermost Black Stripe Formation and lower Blind Fiord Formation suggest that the Ni cycling and isotopic variations were similar to modern Black Sea conditions and consistent with evidence for euxinia at that time^5,20,21^.

### Implications for the end-Permian mass extinction

**Fig. 4:**
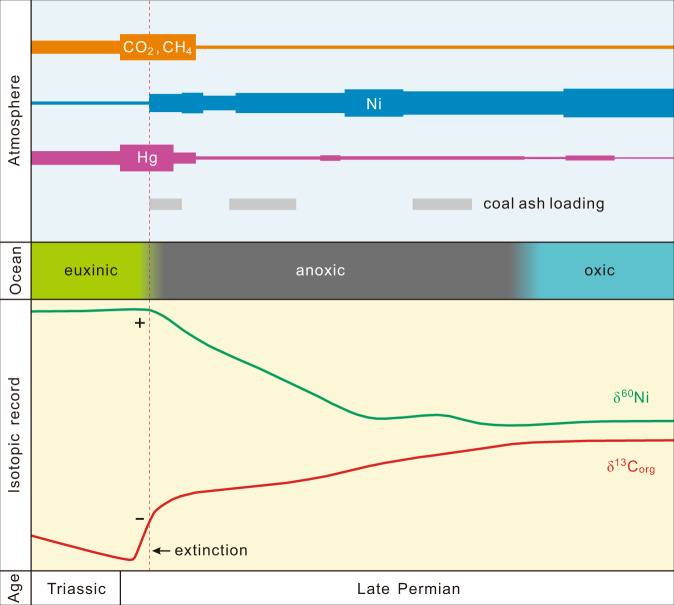
Schematic illustration linking the atmospheric and ocean chemistry changes triggered by the STLIP to the EPME (not to scale). The C-isotope chemostratigraphy and coal ash loading events are from ref. ^5^ and Hg anomalies are from ref. ^11^. Palaeo-redox conditions are from refs. ^21,22^.

Though evidence has previously documented remarkable environmental changes near the EPME horizon, our results demonstrate that environmental perturbations triggered by the STLIP magmatism had begun well before the end-Permian mass extinction, and, more importantly, the pathways to environmental exacerbations leading to the EPME are becoming clearer (Fig. 4). Our δ^60^Ni and Ni content data provide strong evidence for the loading of Ni-rich aerosols into the Sverdrup Basin and a link between eruption of the STLIP, transport of Ni-rich aerosols in the atmosphere, ocean chemistry changes, and mass extinction (Fig. 4). The Buchanan Lake section had a palaeolatitude of 35–40°N from Late Carboniferous to Early Triassic time, and the STLIP eruptions were likely at ~60°N (ref. ^19^) (Fig. 1C). The superbly preserved Buchanan Lake section in the Sverdrup Basin, Canadian High Arctic, therefore, records unparalleled evidence of environmental changes triggered by the STLIP.

Our data indicate that the voluminous release of Ni-rich gases triggered by the STLIP magmatism had changed the Late Permian ocean chemistry well before the EPME (Fig. 4). Nickel is a bioessential trace metal, and the increase of Ni concentrations in the Sverdrup Basin may have increased primary productivity, which is supported by the steady increase of TOC during the same interval^5^ (Fig. 4). The increased primary productivity may have depleted oxygen in the water columns, explaining the shift to increasingly anoxic conditions prior to the main extinction (Fig. 4). Further environmental degradation was accelerated by the main eruption of the STLIP near the extinction horizon (Fig. 4). The spike of Hg/TOC ratios near the extinction horizon at Buchanan Lake suggest toxic Hg conditions^9,11^ (Fig. 4). During the main eruption, the degassing of the sills and volatile-rich sediments of carbonate and coal through contact metamorphism may have liberated extremely high concentrations of iconic greenhouse gases CO_2_ and CH_4_, resulting in rapid global warming, ocean euxinia, and catastrophic climate changes^4–8,13,14,52,53^ (Fig. 4). Sill heating and carbonisation of organic-matter-bearing sedimentary rocks in the Tunguska Basin, as well as the expansion of methanogenic Archaea in the oceans, represent a likely source of ^13^C-depleted carbon, contributing to the pronounced global negative δ^13^C excursion at the extinction horizon^5,7,8,16,52^ (Fig. 4).

Environmental perturbations by voluminous Ni-rich aerosols, coupled with climatic changes driven by greenhouse gases released by the STLIP, such as CO_2_ and CH_4_, likely resulted in continuous environmental catastrophes, leading to the end-Permian mass extinction. There is little doubt that Ni isotope measurements on the Buchanan Lake section open a new window on our understanding of the causal link between the STLIP magmatism and EPME, and further Ni isotopic study of the Late Permian—Early Triassic successions worldwide will improve our understanding of global climatic changes and the most devastating biotic crisis in Earth’s history.

## Methods

### Nickel isotope measurement

Approximately, 100–300 mg of each sample powder was weighted in Savillex screw-top beakers and treated sequentially with distilled 3HF − HNO_3_, HNO_3_ + 3HCl, and HNO_3_. After complete dissolution, solutions were evaporated to dryness at 160 °C and the residues were dissolved in 0.3 N HNO_3_. The Ni concentration of each solution was determined using an Agilent 7700 quadrupole ICP-MS. Based on the measured Ni concentrations, aliquots of sample solutions containing ~1.5 μg Ni were spiked with a ^61^Ni–^62^Ni double spike, in order to reach an optimal spike-sample ratio of 64:36. The spiked solutions were refluxed to ensure sample-spike equilibration before column chemistry.

Separation of Ni from the matrices was achieved using a three-stage, cation exchange chromatography procedure using Bio-Rad 200–400 mesh AG50W-X8 resin^32,37^. The first column separated Fe, Mn, and Cr from Ni using a mixture of 20% 10 M HCl—80% acetone. The second column used 15% 10 M HCl—85% acetic acid to separate Ni from elements such as Mg, Ca, Al, and Ti. The third column further purified Ni by using 0.9 M HNO_3_ to remove Na and K. Four USGS standards, Nod-A-1, BIR-1, BHVO-1, and SCO-1 were processed together with samples for column chemistry. The yield was >85% and the total procedural blank was <10 ng, which is negligible compared to ~1.5 μg of Ni in each sample.

Nickel isotopic compositions were measured using a Nu Plasma II MC-ICPMS at Indiana University with an Aridus II desolvating nebuliser. Four Ni isotopes, ^58^Ni, ^60^Ni, ^61^Ni, and ^62^Ni, were measured simultaneously on Faraday cups. ^57^Fe was also measured and used to correct for interference on ^58^Ni, although this correction was always very small. The background for ^60^Ni was <10^−3^ V, which is negligible relative to sample signals of ~3–4 V. Each sample solution and the four USGS standards were measured 4 times on different days, and the data processing was done with the Matlab code written by S. Romaniello and described in Wasylenki et al.^54^. The Ni isotopic data were reported in the conventional δ notation in per mil relative to NIST SRM 986: [δ^60/58^Ni = (δ^60/58^Ni_sample_/δ^60/58^Ni_SRM986_ − 1) × 1000]. The long-term analytical precision based on the analyses on the pure Ni ICP solution is +0.05‰. The four USGS standards yielded δ^60/58^Ni values of +1.08±0.06‰ for Nod-A-1, +0.17 ± 0.02‰ for BIR-1, +0.05 ± 0.05‰ for BHVO-1, and +0.11 ± 0.04‰ for SCO-1.

## Supplementary information

Supplementary Information

## Data Availability

All data supporting the findings of this study are provided as Supplementary Table 1.
