# Supplementary material for: Nickel isotopes link Siberian Traps aerosol particles to the end-Permian mass extinction
Source: Nat Commun. 2021 Apr 1;12:2024. doi: 10.1038/s41467-021-22066-7 (PMC8016954; doi:10.1038/s41467-021-22066-7)
Supplement: Supplementary file 1 — Supplementary Information [file 41467_2021_22066_MOESM1_ESM.pdf]

SUPPLEMENTARY INFORMATION

Nickel isotopes link Siberian Traps aerosol particles to the end-Permian mass extinction

Menghan Li[1], Stephen E. Grasby[2,3], Shui-Jiong Wang[4], Xiaolin Zhang[1], Laura E. Wasylenki[5], Yilun Xu[1], Mingzhao Sun[6], Benoit Beauchamp[3], Dongping Hu[1], Yanan Shen[1]✉

[1]School of Earth and Space Sciences, University of Science and Technology of China, Hefei 230026, China

[2]Geological Survey of Canada, Natural Resources Canada, Calgary, Alberta T2L 2A7, Canada

[3]Department of Geoscience, University of Calgary, Calgary, Alberta T2N 1N4, Canada

[4]State Key Laboratory of Geological Processes and Mineral Resources, China University of Geosciences, Beijing 100083, China

[5]School of Earth & Sustainability, Northern Arizona University, Flagstaff, AZ 86011, USA

[6]Department of Earth Sciences, ETH Zürich, 8092 Zürich, Switzerland

✉email: yashen@ustc.edu.cn

Supplementary Table 1: The $\delta^{60}Ni$, Ni abundance, TS, TOC, and $\delta^{13}C_{org}$ for the Buchanan Lake section

Sample	Lithology	Depth relative to LPE (m)	$\delta^{60}Ni$	2S.D.	Ni (ppm)	$\delta^{13}C_{org}$	TOC (%)	TS (%)
C-411533-a	black shale	19.4	0.05	0.03	61.7	-27.8	0.47	1.39
C-411530-a	black shale	16.4	0.29	0.08	60.4	-27.6	0.47	0.86
C-411527-a	black shale	13.4	0.20	0.07	43.4	-28.9	0.70	0.87
C-411523-a	black shale	9.4	0.31	0.06	45.7	-30.1	1.04	0.56
C-411518-a	black shale	5.9	0.35	0.04	55.7	-29.9	1.03	0.65
C-411513-a	black shale	4.2	0.17	0.09	37.4	-29.5	0.99	0.59
C-411505-a	black shale	2.6	0.00	0.06	76.5	-29.8	0.71	2.53
C-411503-a	black shale	2.1	0.19	0.10	25.5	-29.3	0.68	1.11
C-411500	black shale	1.5	0.33	0.05	19.7	-29.2	0.71	0.94
C-411498	black shale	1.1	0.19	0.04	32.0	-27.2	0.11	1.95
C-411480	black shale	0.0	0.34	0.11	36.4	-27.8	0.39	3.02
C-411482	black shale	-0.1	0.20	0.04	142.8	-28.7	0.46	3.17
C-411477	black shale	-0.6	0.33	0.02	132.0	-28.1	4.33	1.15
C-411474	black shale	-1.2	0.14	0.04	154.3	-27.0	0.39	1.01
C-411470	black shale	-2.0	0.07	0.05	123.5	-27.6	3.05	0.50
C-411460	black shale	-4.0	0.32	0.08	151.8	-27.6	0.18	0.91
C-411453	black shale	-7.2	-0.01	0.02	164.2	-27.9	0.61	0.49
C-411447	black shale	-10.2	-0.19	0.04	120.0	-28.3	0.16	0.24
C-445083	black shale	-13.0	-0.20	0.07	117.5	-27.7	1.62	0.56
C-445086	black shale	-16.0	-0.03	0.06	164.9	-27.5	3.82	1.04
C-445096	black shale	-26.0	-0.47	0.04	162.9	-27.6	2.22	0.37
C-445100	black shale	-30.0	-0.77	0.01	164.3	-27.4	1.24	12.27
C-445104	black shale	-34.0	-0.87	0.06	210.8	-26.8	1.71	1.15
C-445109	black shale	-40.0	-0.98	0.03	236.9	-25.8	1.62	1.66
C-445113	black shale	-44.0	-0.75	0.04	194.1	-26.2	2.25	1.06
C-445117	black shale	-48.0	-0.61	0.03	187.8	-26.9	4.17	1.62
C-445121	black shale	-52.0	-0.99	0.03	166.6	-25.5	2.04	1.04
C-445127	black shale	-58.0	-0.91	0.01	191.4	-25.7	1.33	0.88
C-445131	black shale	-62.0	-1.09	0.06	157.1	-25.1	1.57	0.60
C-445135	black shale	-66.0	-0.93	0.06	194.6	-25.1	1.22	0.00
C-445139	black shale	-70.0	-0.88	0.05	216.2	-25.7	1.05	1.06
C-445147	black shale	-78.0	-0.89	0.05	247.1	-24.8	0.77	0.27
C-445155	black shale	-86.0	-0.89	0.08	218.3	-25.0	0.77	0.59
